# Microfluidic device for real-time formulation of reagents and their subsequent encapsulation into double emulsions

**DOI:** 10.1038/s41598-018-26542-x

**Published:** 2018-05-25

**Authors:** Jui-Chia Chang, Zoe Swank, Oliver Keiser, Sebastian J. Maerkl, Esther Amstad

**Affiliations:** 1 Institute of Materials, Ecole Polytechnique Fédérale de Lausanne (EPFL), Lausanne, Switzerland; 2 Institute of Bioengineering, Ecole Polytechnique Fédérale de Lausanne (EPFL), Lausanne, Switzerland

## Abstract

Emulsion drops are often employed as picoliter-sized containers to perform screening assays. These assays usually entail the formation of drops encompassing discrete objects such as cells or microparticles and reagents to study interactions between the different encapsulants. Drops are also used to screen influences of reagent concentrations on the final product. However, these latter assays are less frequently performed because it is difficult to change the reagent concentration over a wide range and with high precision within a single experiment. In this paper, we present a microfluidic double emulsion drop maker containing pneumatic valves that enable real-time formulation of different reagents using pulse width modulation and consequent encapsulation of the mixed solutions. This device can produce drops from reagent volumes as low as 10 µL with minimal sample loss, thereby enabling experiments that would be prohibitively expensive using drop generators that do not contain valves. We employ this device to monitor the kinetics of the cell-free synthesis of green fluorescent proteins inside double emulsions. To demonstrate the potential of this device for real-time formulation, we perform DNA titration experiments to test the influence of DNA concentration on the amount of green fluorescence protein produced in double emulsions by a coupled cell-free transcription / translation system.

## Introduction

Emulsion drops are well-suited containers for performing chemical and biochemical reactions under well-defined conditions and in volumes that are significantly smaller than those required to conduct reactions in bulk. This is especially beneficial for high throughput screening assays^1–3^. The accuracy of such assays depends on the degree of control over the composition and concentration of reagents contained inside the drops as well as their size distribution. Drops with a narrow size distribution can be produced using microfluidics^4–6^. These drops have, for example, been employed as containers for drug screening assays^7,8^, to perform polymerase chain reactions (PCR) from viruses^9,10^, or single cells^11–14^, for directed evolution of enzymes^15^, to study the secretion of proteins or other signaling molecules on the single cell level^16,17^, or to identify genes that are responsible for a cellular phenotype^18^. To perform these screening assays, reagents are often pre-mixed before they are injected into the device. Pre-mixing limits drop-based assays to characterizing very slow reactions or late stages of faster reactions because reagents start to react before they are loaded into drops. Moreover, pre-mixing prevents *in situ* changes of the relative reagent concentrations such that only one solution composition can be screened per experiment. A possibility to overcome these shortcomings is the injection of reagents into drops after they have been formed for example through the application of high electric fields^19–24^, the addition of chemicals that destabilize drops^25^, or the use of gas bubbles to spatially separate adjacent drops^26^. Using AC electric fields, the concentration of reagents in drops can also be oscillated through consecutive fusion and fission processes^27^. However, the number of different reagents that can be controllably added to intact drops is limited. Moreover, it is difficult to accurately and continuously vary the concentration of injected reagents.

The reagent concentration can be gradually changed by co-flowing two fluids under laminar conditions; in this case, the reagent exchange is diffusion limited^28–31^. However, because mixing relies on diffusion, the spatio-temporal control over the solution composition is poor. This control can be improved if mixing is enhanced, for example by introducing turbulences into the fluid flow using structured microchannels^32,33^ or active mixers, such as micropumps, or micromixers^34^. However, it always takes some time to equilibrate injection flow rates especially if multiple fluids are involved. Thus, even with mixing features being implemented, it is difficult to controllably and continuously change the concentrations of different reagents with a high temporal resolution.

The concentration of reagents can be changed over a wide range and on very short time scales if microfluidic channels are equipped with integrated pneumatic valves that can be rapidly opened and closed^35^. The large-scale integration of micromechanical valves^36^ fabricated by soft-lithography^37^ has led to the development of a wide range of microfluidic devices and their applications in areas such as single cell analysis^38^, protein biochemistry^39^, drug discovery^40^, systems biology^41,42^, synthetic biology^43,44^ and molecular diagnostics^45^. These valves allow the formation of a train of alternating plugs of different types of miscible liquids that can subsequently be mixed. The length of the plug of each fluid scales with the duty cycle, corresponding to the pulse width of the corresponding valve divided by the entire cycle period, and can be adjusted *in situ* and in real-time. Hence, the relative reagent concentrations can be varied over a wide range within a single experiment by gradually changing the duty cycle. This method of mixing fluids, based on pulse width modulation (PWM), has been implemented in microfluidic devices^46,47^ and is often employed to synthesize biopolymers, to study the influence of their composition on their function, and to test the effect of certain molecules on cell behavior^48–53^. Recent advancements of this technology enable independent injection of up to six different reagents and changing their concentrations by up to five orders of magnitudes^35^. This level of compositional control over such a wide concentration range is difficult to achieve with co-flowing fluids. The ability to rapidly and controllably change the concentration of reagents contained in the cores of double emulsions by PWM would open up new possibilities for high throughput screening assays. However, to the best of our knowledge, pneumatic valves have never before been implemented into microfluidic flow focusing drop makers and consequently PWM has thus far not been used to control the concentration of reagents contained in the cores of double emulsions.

In this paper, we present a microfluidic flow focusing drop maker that has three inlets for reagents, each of them controlled by a pneumatic valve. This device allows real-time mixing of multiple reagents using PWM, and encapsulates the resulting solution containing a homogeneous mixture of reagents in double emulsions. The pneumatic valves provide an additional benefit: they enable encapsulation of liquids with volumes as low as 10 µL at an efficiency approaching 100%. To test the performance of the device, we encapsulate lysates that synthesize green fluorescent protein (GFP) inside double emulsion drops. We demonstrate the potential of the device by conducting DNA titration assays to measure the influence of DNA template concentration on the amount of GFP produced in double emulsion drops.

## Results

**Figure 1:**
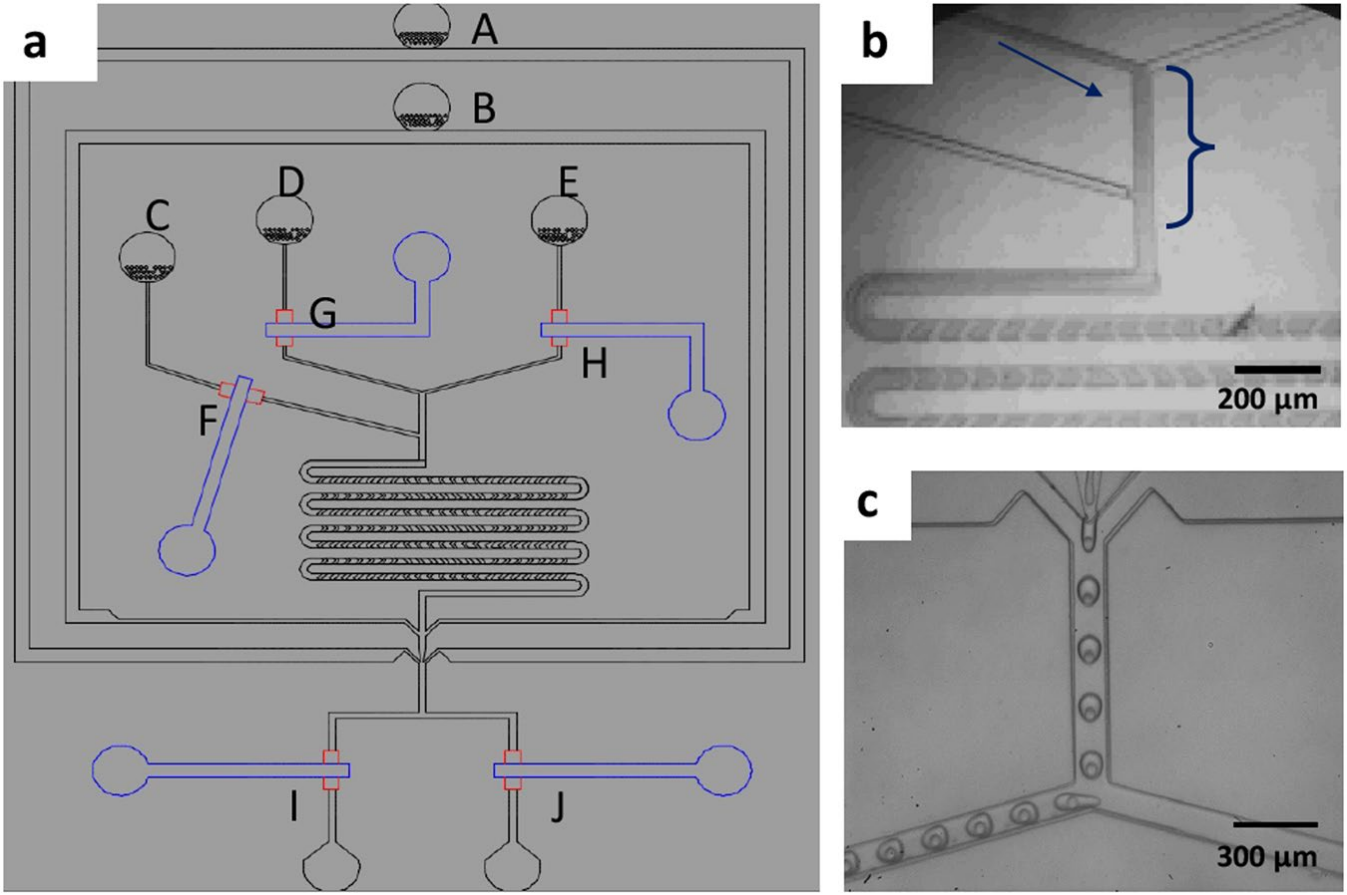
Schematic illustration of the microfluidic device (**a**) Overview of the device with the inlet for (A) the outermost aqueous phase, (B) the oil phase and (C–E) the three innermost aqueous phases. On top of each inlet for the innermost phase is a pneumatic valve that enables controlling the fluid flow (F–H); the control lines are indicated in blue. Drops exit the device through one of the two outlet channels that also contain pneumatic valves (I,J). (**b**) Optical micrograph of an operating device where an aqueous phase and a dye are alternatingly injected using PWM. (**c**) Optical micrograph of double emulsions that are collected through the left outlet.

We fabricate microfluidic devices using soft lithography^37^. Devices contain three inlets for aqueous solutions that form the inner phase, one inlet for the oil that constitutes the middle phase, and one inlet for an aqueous phase that forms the surrounding liquid. To control the fluid flow in the three inlets for the innermost phase, we introduce pneumatic valves on top of these channels, as schematically shown by the blue features in Fig. 1a^36^. The valves enable alternating flow of fluids into the main channel using pulse width modulation, as exemplified in the optical micrograph in Fig. 1b^35,54,55^. To accelerate the mixing of the sequentially injected reagents, herringbones are introduced to the serpentine-like section of the main channel located between the inlets for the innermost phases and that for the oil phase^33^, as schematically shown in Fig. 1a. To separate reagent-loaded drops from empty ones, the device has a T-junction used as a sorting unit, as shown schematically in Fig. 1a and in the optical micrograph in Fig. 1c. The flow of double emulsions is again controlled with pneumatic valves that change the hydrodynamic resistance of the outlet channels.

**Figure 2:**
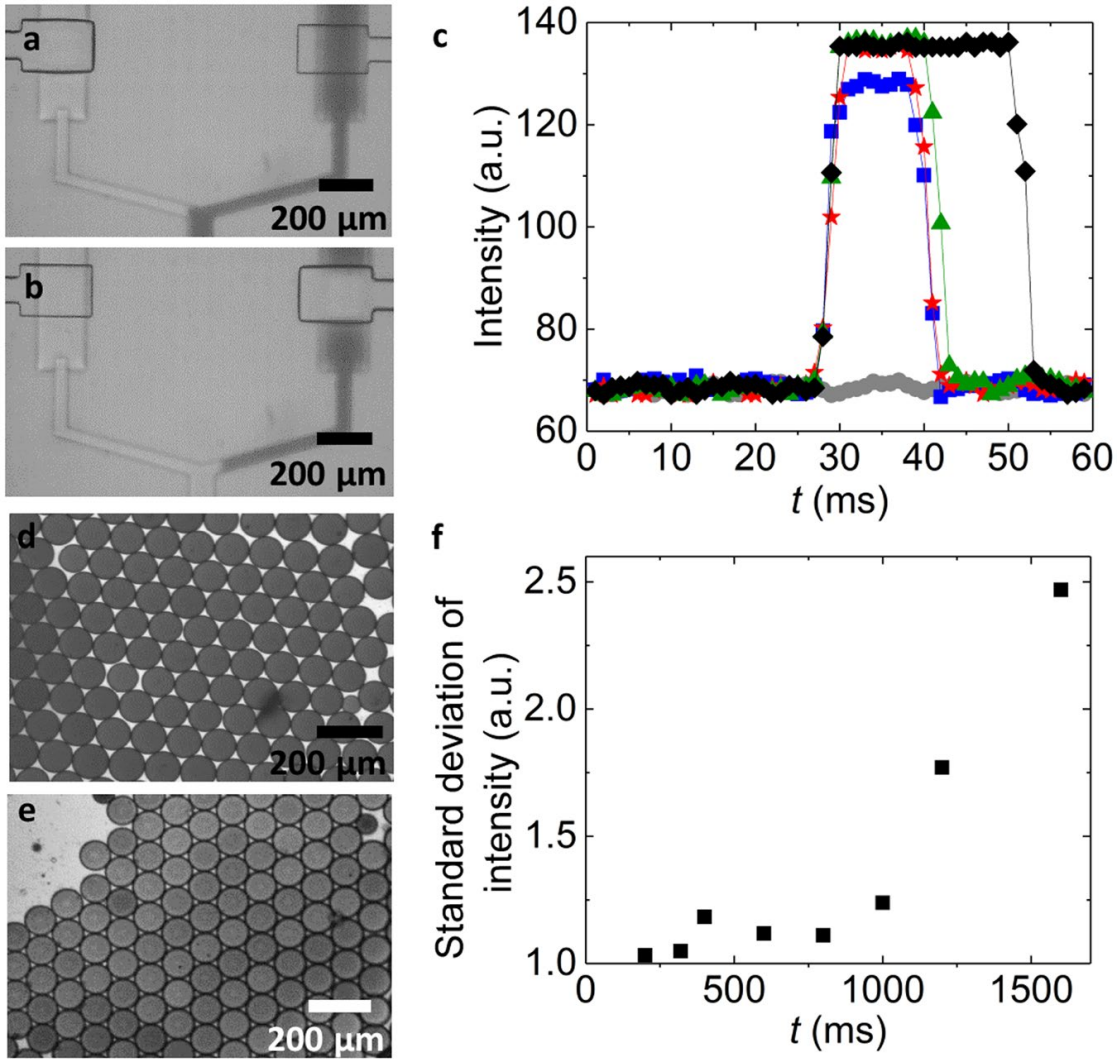
Characterization of the pneumatic valves. (**a**,**b**) Optical micrographs of a pneumatic valve that is (**a**) opened and (**b**) closed. (**c**) The intensity of the colorant measured underneath the valve as a function of time for duty cycles of 50% and cycle period of 40 ms (), 50 ms (), 60 ms (), 80 ms (), and 100 ms (♦). (**d**,**e**) Optical micrographs of drops formed with a cycle period of (**d**) 200 ms and (**e**) 1600 ms. (**f**) The standard deviation of the dye intensity in drops as a function of the cycle period.

To precisely vary the relative amounts of reagents injected into the main channel, the lengths of the sequentially injected plugs must be controlled. This can be achieved by tuning the duty cycles of the valves that control the flows of the inner phases. Valves close if their control channels are pressurized such that the valves are pressed towards the bottom of the fluid channel. We employ software-controlled solenoid valves to pressurize and depressurize the control channels^56–58^. To determine the response time of the microfluidic valves, we use a colored fluid and quantify the intensity profile across the fluid channel underneath the valve as a function of the pulse width, which is the time the valve is open. If the valve is open, the entire inlet channel underneath the valve is colored, as shown in the optical micrograph in Fig. 2a; in our device, the inlet channel section below the valve is 200 µm wide. If the valve is closed, the fluid is pushed aside such that this section becomes transparent, as shown in Fig. 2b. If we keep the duty cycle constant at 50% and set the cycle period to 40 ms, no fluid can pass, as demonstrated by the flat curve composed of grey circles in Fig. 2c. If we increase the cycle period to 60 ms, some fluid passes and even more fluid passes if the cycle period is increased to 80 ms, as indicated by the increased peak intensity of the curves with the blue squares and red stars in Fig. 2c. A further increase in the cycle period broadens the intensity peak but does not increase its amplitude any more, as shown in Fig. 2c. These results indicate that the minimum time each valve must be open and hence the minimum pulse width is 40 ms, corresponding to a minimum cycle period of 80 ms for duty cycles of 50%. This minimum pulse width is limited by the time required to depressurize the control channel and sets a lower limit to the size of plugs that can be formed. This plug size also depends on the fluid flow rate that we set to 250 µL/h; in this case, the minimum plug volume is 3.5 nL. To test if we can control the concentration of reagents in drops, we vary the pulse width of the valve that controls the flow of the color from 50 ms to 200 ms. Indeed, the color of the resulting drops increases linearly with a change in pulse width and hence, with increasing duty cycle, as shown in Figure S1. These results indicate that we can control the composition of the drops by tuning the duty cycles of the valves.

A key feature of the device is its ability to *in situ* change the composition of encapsulated reagents in real-time and with high precision. This degree of control is only possible to obtain, if adjacent plugs that contain different reagents are homogeneously mixed before the solution is compartmentalized into drops. The time required to achieve a homogeneous mixing of reagents increases with the plug length because their diffusion length increases. To quantify the maximum length of plugs that our device can process into a homogeneous mixture before the solution is broken into drops, we employ two liquids, water and a water-soluble dye, as an inner phase. The lengths of the plugs are varied by changing the cycle periods while we keep the duty cycles constant at 50%. If the cycle period is short such that the liquid plugs are small, the two aqueous phases are fully mixed when the solution reaches the water-oil junction. As a result, the intensity of the 70 µm diameter drops that form at this junction is homogeneous, as indicated in Fig. 2d. However, if we increase the cycle period above a certain value where plugs become too long, the composition of the solution at the oil-water junction starts to vary over time such that the intensity of the resulting drops is heterogeneous, as shown in Fig. 2e.

To quantify the maximum length of aqueous plugs that can be fully mixed in our device, we measure the standard deviation of the drop intensity as a function of the cycle period; these experiments are performed using liquids whose viscosity is close to that of water. If the cycle period is below 800 ms, the drop intensities are uniform, as shown in the optical micrograph in Fig. 2d and summarized in Fig. 2f. By contrast, if the cycle period is increased above this value, the intensity becomes heterogeneous, as shown in the optical micrograph in Fig. 2e and summarized in Fig. 2f. These results indicate that the maximum cycle period where reagents are still homogeneously mixed before the solution is broken into drops is 800 ms; this corresponds to a maximum pulse width of 400 ms and a maximum plug volume of 35 nL. Hence, in our device, we can vary the plug volumes from 3.5 to 35 nL. However, these are not fundamental limits. The dynamic range of the device could be increased by prolonging the mixing unit, or by altering the channel dimensions.

Droplet microfluidics allows encapsulation of reagents with high efficiency. However, in most cases, 50 and 100 µL reagents are lost during device start-up. This fluid loss is no problem if reagents are inexpensive and available in large quantities. However, some biological assays involve expensive reagents or samples that are only available in very small volumes. To process these solutions with microfluidic drop makers, sample losses should be minimized. The loss of reagents can be reduced essentially to zero if microfluidic devices contain pneumatic valves: using these valves, the device can be initialized with an aqueous solution while the channels containing expensive or rare samples are closed. These reagent-containing channels are opened only when the device runs stably, such that no reagents are lost during start-up. To separate reagent-loaded drops from empty ones, we again employ pneumatic valves that are incorporated into the collection channels. To test the performance of these valves, we form aqueous single emulsion drops and collect them through the right outlet. To avoid that drops break while they pass the channel underneath the valve, that is only 14 µm tall and hence acts as a constriction, we increase this height to 20 µm^59^. These valves do not completely close the outlet channels even if they are fully pressurized. Instead, they decrease the height of the channel, thereby increasing its hydrodynamic resistance. As a result of the high hydrodynamic resistance of one of the outlet channels, the vast majority of the fluid flows through the other outlet channel whose valve is open and hence, whose hydrodynamic resistance is much lower. We exemplify this behavior by closing the valve of the left collection channel and opening the one of the right channel. Indeed, drops remain intact if they pass these modified valves. Using these valves, the flow direction of drops can be switched from right to left, as indicated in the optical micrograph in Figure S2a, demonstrating that these modified valves are well suited to sort drops.

To maximize the accuracy of the sorting, we close the valve of the right channel 10 ms before we open the valve of the left channel. This procedure reduces pressure differences between the two collection channels and therefore allows the drops to flow into the desired collection channel as soon as the corresponding valve is opened. If the injection rate is below 600 µL/h, drops can be sorted without any loss, as shown by the optical time-lapse images in Figure S2a. Under these operating conditions, no sample is lost whatsoever. By contrast, if the injection rate exceeds 600 µL/h, some drops split at the sorting junction during the switching operation, as shown in the optical time laps images in Figure S2b, resulting in some sample loss. The volume loss increases with increasing injection rate, as summarized in Figure S2c. Hence, if appropriate flow rates are used, our device enables a loss-free compartmentalization of fluid volumes as small as 10 µL into well-defined drops and the separation of reagent-containing drops from empty ones.

**Figure 3:**
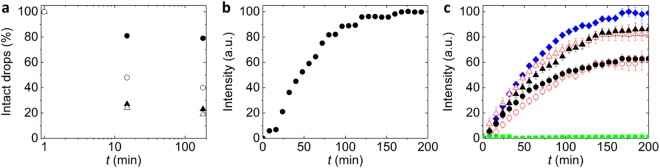
Synthesis of GFP in drops. (**a**) The percentage of drops that remains intact as a function of the incubation time for single emulsion drops loaded with 30 mM (△) and 4 mM 3-PGA (▲) and double emulsion drops containing 30 mM (○) and 4 mM 3-PGA (●). (**b**) The normalized fluorescence intensity measured in double emulsion drops loaded with PURE as a function of the incubation time. (**c**) The normalized fluorescence intensity of lysate solutions in bulk (), in single emulsion drops loaded with lysates through PWM (▲) and with pre-mixed lysates (), and double emulsion drops loaded with lysates through PWM (●) and with pre-mixed lysates () as a function of the incubation time. As a control, lysate extract was loaded into drops in the absence of DNA ().

To test the performance of our device, we employ it to produce drops loaded with reagents that form proteins through cell-free transcription / translation. If drops are used as containers to screen synthesis conditions, they must be stable during the reaction. Biological drop-based screening assays are most frequently performed using single emulsion drops that are dispersed in a fluorinated oil^18,60^; these drops are often stabilized with a perfluorinated triblock copolymer surfactant^61^. To test if such aqueous drops remain intact during the synthesis of proteins, we loaded them with lysates^62,63^ and a solution containing 30 mM 3-PGA and incubate them at 29 °C for 3 h. We observed that most single emulsion drops coalesce during their incubation such that the resulting sample is polydisperse. This coalescence limits the use of drops for many screening applications. We assign the limited drop stability to the high concentration of ions present in the emulsion drop^64^. To test if we can increase drop stability, we decrease the concentration of 3-PGA to 4 mM. However, also in this case, single emulsion drops tend to coalesce albeit to a smaller extent, as summarized in Fig. 3a and shown in the fluorescence micrographs in Figure S3a and b.

To screen synthesis conditions inside drops, they should be stable against coalescence. The coalescence of single emulsion drops is likely caused by ions that are in close proximity to the surfactants or the drop interfaces. If this is the case, double emulsions should be more stable against coalescence because their outer liquid-liquid interface is spatially separated from ions by the oil shell. Indeed, the percentage of intact double emulsions is significantly higher than that of single emulsion drops, even if a solution containing 30 mM 3-PGA is used as an innermost phase, as summarized in Fig. 3a. These results indicate that double emulsions are potentially more suitable screening vessels if reactions are conducted at high salt concentrations and elevated temperatures because they are more stable against coalescence.

To test if we can use these double emulsions as picoliter-sized vessels to produce proteins through cell-free synthesis, we employ PURE, a cell-free transcription / translation reaction mixture generated from purified components^65^. PUREexpress is now commercially available (NEB) but is a relatively expensive reagent with a cost of over 1 USD per µL. Therefore, it is beneficial to only consume small volumes. Yet, small volumes are difficult to handle with standard microfluidic drop makers where volumes between 50 and 100 µL can easily be lost during start-up. To prevent sample loss during start-up of our device, deionized water is injected through inlet C. To ensure proteins are only produced inside double emulsion drops and do not start to form in the bulk solution prior to their injection in the device, we do not pre-mix reagents but co-inject two solutions containing complementary reagents. When the device runs stably, valve F that controls the flow of pure water is closed, and the two solutions are sequentially flowed using PWM with duty cycles of 50% and a cycle period of 100 ms. One aqueous solution contains PURE and is injected through inlet D. The other aqueous solution contains DNA and is injected through inlet E. The reagents are mixed in the channel section that contains herringbones and the resulting solution is employed to form the cores of 65 µm diameter double emulsion drops. These drops are incubated at 29 °C for 3 h and the synthesis kinetics of GFP is measured by acquiring a fluorescent micrograph every 8 min. Within 2 h fluorescence reaches a plateau as shown in the fluorescence intensity trace in Fig. 3b. These experiments demonstrate the potential of our device for the economic use of expensive reagents such as PUREexpress.

Double emulsions are significantly more stable than single emulsions. Nevertheless, a large fraction of the cores containing 30 mM 3-PGA merges with the surrounding aqueous phase such that all the encapsulants are released, as exemplified in Fig. 3a. To increase the fraction of intact double emulsion drops, we reduce the 3-PGA concentration to 4 mM. This reduction in 3-PGA concentration significantly increases the percentage of intact double emulsions, as exemplified in the fluorescence micrographs in Figure S3c,d and summarized in Fig. 3a. Hence, solutions containing 4 mM 3-PGA are employed for the following experiments.

To investigate the kinetics of synthesized GFP, we quantify the fluorescence intensity of each drop. Encapsulation of lysates into 65 µm diameter drops does not alter the kinetics of GFP synthesis, as summarized in Fig. 3c. However, the amount of GFP produced in double emulsions is significantly lower than that produced in single emulsion drops and in bulk, as indicated by the blue diamond in Fig. 3c. In our experiments, single emulsion drops contain approximately 0.55 µM functional GFP whereas double emulsion drops contain only approximately 0.39 µM functional GFP, as quantified using a calibration curve and summarized in Figure S4. This difference might be caused by a partial leakage of reagents through the shell of double emulsions, by analogy to the crosstalk observed between aqueous drops that are dispersed in perfluorinated oils^66^. Nevertheless, there is a significant amount of functional GFP synthesized in double emulsion drops, indicating that they can be used to screen effects of synthesis conditions on the amount of functional GFP produced.

**Figure 4:**
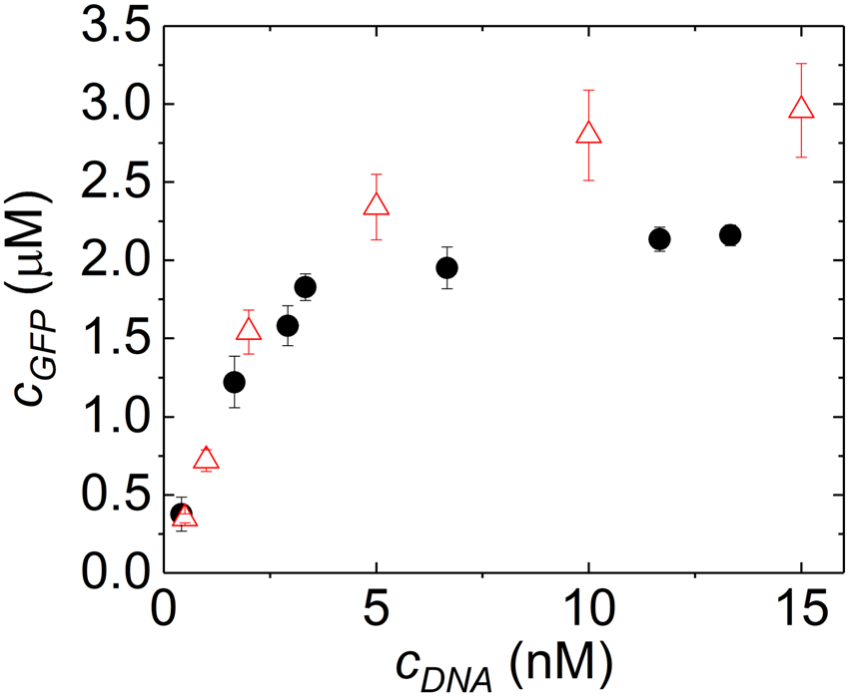
*In situ* DNA-titration. The influence of the DNA concentration on the amount of GFP synthesized inside double emulsions (●) and in bulk (). For GFP synthesized in double emulsions, the DNA concentration was varied *in situ* by pulse width modulation.

One of the main features of our microfluidic device is its ability to change the concentrations of reagents contained in double emulsion drops in real-time and with high accuracy. To exploit this feature, we perform DNA titration experiments: Water is injected through inlet D, an aqueous solution containing lysate extracts from *E*. *coli*, 30 mM 3-PGA, and an energy source through inlet C, and an aqueous solution containing 15 nM DNA through inlet E. The total cycle period is kept constant at 400 ms while the duty cycle of valve E is varied from 10% to 80% in four steps. Using this procedure, we screen four DNA concentrations, 13.3, 11.7, 6.7, and 1.7 nM, within a single experiment that consumes as little as 10 µL of lysates and similar volumes of the energy solution. From these reagents, we produce approximately 100 drops for each DNA concentration. We repeat the same experiment using a solution containing 7.5 nM DNA to screen four additional DNA concentrations. The resulting double emulsions are incubated for 3 h at 29 °C and their intensity is quantified using fluorescent microscopy. The amount of protein produced in a drop increases with increasing amounts of DNA up to a concentration of 6 nM and levels off thereafter, as shown by black circles in Fig. 4. A similar trend is seen in the experiments performed in bulk, as shown by red triangles in Fig. 4. However, using drops, we obtain a 25-fold improved statistics while consuming 8-fold less reagents than if the screening is performed in bulk. These results demonstrate the potential of our device to screen different reaction conditions with very low volumes of reagents.

## Conclusions

We present a microfluidic drop maker that allows real-time formulation of three solutions using pulse width modulation and processes the resulting mixture into cores of double emulsion drops. The device allows processing solutions with volumes as low as 10 µL into cores of double emulsions without suffering from sample loss. To demonstrate the potential of the device to characterize influences of the reagent concentrations on the products synthesized in double emulsion drops, we synthesize GFP using a cell-free reaction and titrate DNA template concentrations. The economic use of expensive reagents that includes the ability to change the reagent composition within a single experiment are of particular importance for the production of expensive biomolecules and for screening and characterization of samples that are only available in very small quantities. Hence, this device might open up new possibilities to screen synthesis conditions for reactions that involve expensive or rare reagents.

## Materials and Methods

### Materials

GFP is synthesized by mixing an aqueous solution containing cell-free reagents and an energy solution. The cell-free reagent solution contains lysate extracted from *E*. *coli* and GFP DNA templates. The energy solution is composed of water containing 10.5 mM magnesium glutamate, 100 mM potassium glutamate, 0.25 mM dithiothreitol (DTT), 1.5 mM of each amino acid except leucine, 1.25 mM leucine, 50 mM HEPES, 1.5 mM adenosine triphosphate (ATP), and guanosine-5’-triphosphate (GTP), 0.9 mM cytidine triphosphate (CTP) and uridine triphosphate (UTP), 0.2 mg/mL tRNA, 0.26 mM coenzyme A (CoA), 0.33 mM nicotinamide adenine dinucleotide (NAD), 0.75 mM cyclic adenosine monophosphate (cAMP), 0.068 mM colonic acid, 1 mM spermidine, 2% PEG-8000, 4 mM 3-Phosphoglyceric acid (3-PGA).

### Fabrication of Microfluidic Device

The microfluidic device is made of poly(dimethylsiloxane) (PDMS) using soft lithography^37,67^. It contains five inlets, one for the outer phase, one for the middle phase, and three for the inner phases. The fluid flow of the inner fluids is tuned with control valves through which air is injected at a pressure of 25 psi to close the pneumatic valves located on top of the respective fluid channels. The three inlets for the inner phases lead into a 50 µm wide channel that contains herringbone structures to enhance the mixing of the reagents. Downstream the herringbone structures, the main channel is intersected by two inlets for oil that forms the shell of the double emulsions. The oil and the aqueous solution are processed into double emulsion drops at a 3D junction^67^ that leads into a 100 µm wide collection channel.

The maters used for the bottom part of the device containing the liquid channels is fabricated from two layers of negative photoresist, SU-8; the first layer is 14–20 µm tall, the second layer is 100 µm tall. The masters employed to fabricate the top part of the device is made of three layers of photoresist: The first layer is 14 μm tall and composed of a positive photoresist, AZ9260, each of the second and third layer is 20 µm tall and composed of a negative photoresist, SU-8.

The microfluidic device is made from Sylgard 184 PDMS (Dow Corning). The three parts are joined through reactive bonding: To fabricate the top part of the device, we employ a base: crosslinker ratio of 1: 5, the middle part is made at a base: crosslinker ratio of 1: 20, and the bottom part at a base: crosslinker ratio of 1: 10^36^. The middle part must be thin to ensure that the valves are sufficiently flexible to close the fluid channels if the control channels are pressurized. To control the thickness of the middle part, we spin coat PDMS to form a 100 µm thick layer. PDMS is cured at 80 °C for 25 minutes. The top and middle parts are aligned and bonded by incubating them at 80 °C for 2 hours. The resulting part is removed from the mold and bonded to the bottom part using oxygen plasma followed by incubation at 65 °C for 12 hours. The resulting devices have 100 µm tall control channels. The inlet liquid channels are 20 µm tall and lead into the three dimensional junction where the outermost liquid phase meets the main channel; at this junction the channel height increases to 60 µm.

To produce water-oil-water double emulsions, the part upstream the junction where the outermost phase flows into the main channel must be hydrophobic whereas the main channel further downstream must be hydrophilic. To render the top part of the device hydrophobic we inject fluorinated oil (Novec 7500, 3 M, MN) containing 1 vol% trichloro1H,1H,2 H,2H-perfluorooctyl)silane (Sigma-Aldrich, MO) into this section of the device. To render the remaining part of the device hydrophilic, the surfaces are treated with an aqueous solution containing 2 wt% poly(diallyldimethylammonium chloride) and 1 M sodium chloride.

Fluids are injected into the device through polyethylene tubing (PE/5, Scientific Commodities Inc., AZ) using syringe pumps.

### Encapsulation of cell-free reagents

We employ an aqueous solution containing 10 wt% of poly(vinyl alcohol) (PVA), *M*_*w*_ 13 000–23 000 Da, 87–89% hydrolyzed, as an outer phase, a perfluorinated oil, HFE7500 (Novec 7500, 3 M, MN), containing 1 wt% of surfactant^61,64^ as a middle phase, and an aqueous phase as an inner phase. To initialize the device, we use deionized water as an inner phase. Once the device runs stably, we close the valve F that controls the fluid flow of the deionized water. Simultaneously, the two other valves, D and E, are alternatingly opened to inject the aqueous solutions containing the reagents from the two other inlets for the inner phase. Within one experiment, we inject 10 µL of an aqueous solution containing lysate extract solution through inlet D and 10 µL of an aqueous solution containing DNA and energy source through inlet E. Thereby, we keep the duty cycles constant at 50% and the total cycle period is 100 ms.

### DNA titration

To perform DNA titration experiments, we employ three different aqueous phases as inner phases: Inlet E contains lysate with an energy source, inlet D contains an aqueous solution with 15 nM of DNA, and inlet C contains pure water. We change the DNA concentration, without changing the concentration of any other reagent, by varying the duty cycles of valves G and H that control the flow of the aqueous solutions containing pure water and DNA respectively. The cycle period is 400 ms and the duty cycles vary from 10% to 80% in four steps. To enlarge the range of DNA concentrations that are screened, we repeat the same experiment but inject an aqueous solution containing 7.5 nM of DNA through inlet D. Drops are subsequently incubated at 29 °C for 3 h. During this incubation, we monitor the formation of green fluorescence protein using fluorescence microscopy where one image is acquired every 8 minutes.

### Analysis of the formation of green fluorescence proteins

The formation of GFP is quantified using fluorescence micrographs. The average fluorescence intensity of each drop is quantified and normalized for its size and shape. To correct for lensing effects that occur at the drop interfaces, we perform control experiments where the fluorescence intensity of solutions containing known amounts of fluorescein is measured in bulk, in single emulsion, and double emulsion drops. Lensing effects increase the fluorescence intensity in single emulsions by 11%, compared to the bulk and in double emulsions by 15%. We correct for these lensing effects and convert the fluorescence intensity into a protein concentration for each drop using a calibration curve measured in bulk. For each data point reported, we analyze the protein concentration of at least 25 drops to calculate the error bars.

## Electronic supplementary material

Supporting Information
