# Supplementary material for: Microfluidic device for real-time formulation of reagents and their subsequent encapsulation into double emulsions
Source: Sci Rep. 2018 May 25;8:8143. doi: 10.1038/s41598-018-26542-x (PMC5970246; doi:10.1038/s41598-018-26542-x)
Supplement: Supplementary file 1 — Supporting Information [file 41598_2018_26542_MOESM1_ESM.pdf]

Supporting Information

Microfluidic device for real-time formulation of reagents and their subsequent encapsulation into double emulsions

Jui-Chia Chang,[a] Zoe Swank,[b] Oliver Keiser,[a] Sebastian Maerkl,[b*] Esther Amstad[a*]

[a.] Institute of Materials, Ecole Polytechnique Fédérale de Lausanne (EPFL), Lausanne

[b.] Institute of Bioengineering, Ecole Polytechnique Fédérale de Lausanne (EPFL), Lausanne

Influence of duty cycle on the amount of reagent contained in drops

To determine if the amount of reagent that is contained in drops scales with the duty cycle of the valve, corresponding to the fraction of time the valve is open relative to the entire cycle period, we employ an aqueous solution containing fluoresceine. We co-inject this solution with an aqueous solution that does not contain any fluorophore. We keep the pulse width of the valve controlling the flow of the pure aqueous phase constant at 50 ms and vary that of the valve controlling the flow of the fluorescent phase between 50 and 200 ms. Indeed, the measured fluorescence linearly increases with the duty cycle, as shown in Figure S1.

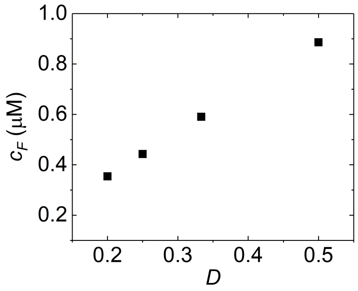

Figure S1: Influence of the duty cycle (*D*) on the concentration of fluoresceince, c_F, contained in drops.

Drop sorting

To separate reagent-containing drops from empty ones, we introduce a T-junction downstream the drop generation junction. By pressurizing the left control channel of the sorting unit, the left valve partially closes the channel such that its hydrodynamic resistance increases and drops flow into the right outlet. To switch the direction of the fluid flow, we close the right valve 10 ms before we open the left one. If the fluid flow upstream the T-junction does not exceed 600 µL/h, drops follow the fluid flow and remain intact even when the fluid flow switches direction, as shown in the time lapse optical micrographs in Figure S2a. By contrast, if the injection flow rate exceeds 600 µL/h, some drops split at the T-junction while the direction of the fluid flow is changed, resulting in some loss of reagents, as shown in the time lapse optical micrographs in Figure S2b. The amount of sample that is lost increases with increasing injection rate, as sumarized in Figure S2c.

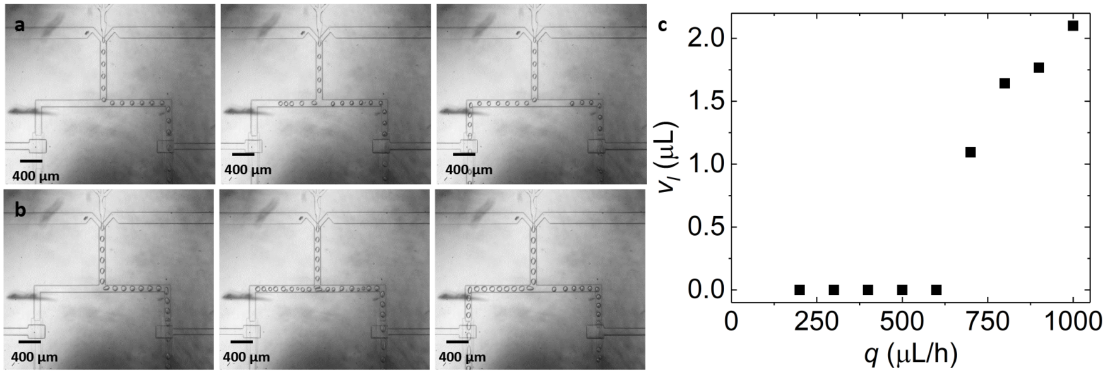

Figure S2: Drop sorting. Time lapse optical microscopy images illustrating the change in the flow direction of drops if the total injection speed is (a) 600 µL/h and (b) 900 µL/h. (c) The amount of fluid lost during the switching process (v_l), as a function of the injection flow rate, q, is shown.

Synthesis of green fluorescence proteins in drops

To test if we can track the formation of GFP inside drops, we form monodisperse single emulsion drops with a diameter of 70 µm that are loaded with lysates and 4 mM of 3-PGA. Even though the PGA concentration in these drops is almost an order of magnitude below the PGA concentration typically used, most of the drops coalesce, as indicated by the high polydispersity of drops incubated at 29°C for 30 min, shown in Figure S3a and the even higher polydispersity of drops after they have been incubated at this temperature for 3 h, as shown in Figure S3b. Coalescence of drops hampers their use for screening assays. We expect the high concentration of ions that are in close proximity to the liquid-liquid interface to deter drop stability. To test this expectation, we produce double emulsion drops containing lysates and 4 mM 3-PGA in their core; the outer liquid-liquid interface of these drops is separated from ions by their oil shell. Indeed, double emulsions are much more stable against coalescence, as indicated by their narrow size distribution after they have been incubated at 29°C for 30 min, as shown in Figure S3c and 3 h, as shown in Figure S3d.

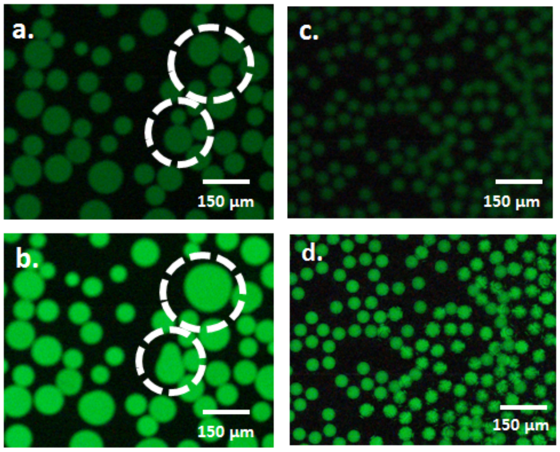

Figure S3: Synthesis of GFP in drops with 4 mM 3-PGA. (a-d) Fluorescence micrographs of (a, b) single emulsion and (c, d) double emulsion drops loaded with lysates an incubated at 29°C for (a, c) 30 min and (b, d) 3 h.

Quantification of green fluorescent protein concentrations

To quantify the amount of GFP produced in single and double emulsion drops we measure a calibration curve in bulk. The amount of GFP produced in solutions containing 4 mM 3-PGA is approximately 50% lower compared to solutions containing 30 mM, as summarized in Figure S4. By contrast, the kinetics of the GFP production is not affected by the concentration of 3-PGA, as shown in Figure 3c in the main paper.

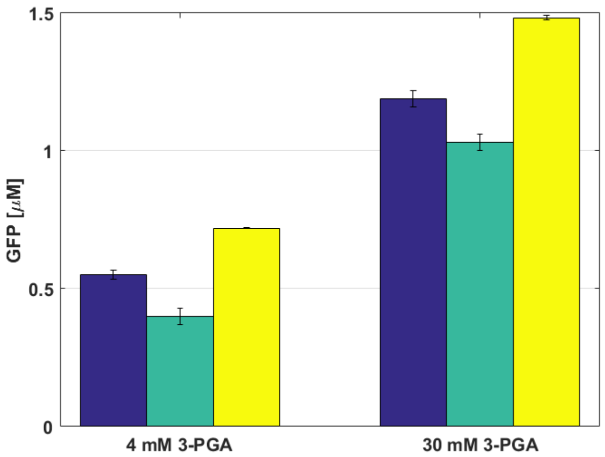

Figure S4: Concentration of GFP synthesized in 65 µm drops containing 4 mM and 30 mM 3-PGA. GFP is synthesized in single emulsions (blue), in double emulsions (green), and in bulk solution (yellow).
